# Interleukin-4 receptor targeted immunotherapy of human bladder cancer in animal models

**DOI:** 10.1186/2051-1426-2-S3-P184

**Published:** 2014-11-06

**Authors:** Bharat H Joshi, Akiko Suzuki, Pamela Leland, Samir Lababidi, Frederick Varrichio, Robert Kreitman, Raj K Puri

**Affiliations:** 1 Division of Cellular and Gene Therapies, Center for Biologics Evaluation and Research, FDA, Bethesda, USA; 2 CBER, FDA, USA; 3 CBER, FDA, Silver Spring, USA; 4 CBER, FDA, Wakefield, USA; 5 NCI, NIH, USA

## 

Previously, we have demonstrated that Interleukin-4 (IL-4) receptor alpha (IL-4Rα) is overexpressed in bladder cancer biopsy specimens and its expression level correlates with the grade and stage of disease. Based on these observations, it is proposed that IL-4Rα is a prognostic biomarker for bladder cancer. To target IL-4Rα, we have developed a recombinant chimeric fusion immunotoxin, which consists of circularly permuted IL-4 and truncated *Pseudomonas *exotoxin (IL-4-PE) [1]. Here we demonstrate that IL-4-PE is highly cytotoxic to eight bladder cancer cell lines *in vitro*. The cytotoxicity by IL-4-PE was mediated in a concentration dependent manner and this cytotoxicity was receptor specific as excess IL-4 inhibited cytotoxicity mediated by IL-4-PE. IL-4-PE immunotoxin also killed bladder cancer colonies in a concentration dependent manner in a clonogenic assay. We developed three subcutaneous tumor models in athymic nude mice using three different bladder cancer cell lines (UM-UC-3, SW780 and 5637), which are sensitive to IL-4-PE at a variable degree. These mice were treated with 50 μg/kg, 100 μg/kg of IL-4-PE immunotoxin or vehicle-control intratumorally and monitored for tumor growth and survival. IL-4-PE effectively caused regression of tumors by 70% in all three tumor models compared to vehicle control mice. Responding animals showed complete regression of tumors in 58% of mice at the highest dose in UM-UC-3 tumor model and 54% in SW780 tumor model. Overall, all responding animals showed >8 week longer survival compared to control mice. IL-4-PE immunotoxin at both doses did not show any visible toxicity when administrated intratumorally. Similar safety profile has been observed in the clinic when IL-4-PE was administered intratumorally in glioma trial [2]. Taken together our results demonstrate that IL-4Rα in bladder cancer is a prognostic biomarker and in addition it provides an excellent target for immunotherapy. Additional studies are ongoing to target IL-4Rα with other immunotherapeutic approaches such as cancer vaccines and adoptive cell transfer immunotherapy.
